# Westminster Hospital

**Published:** 1895-06-29

**Authors:** 


### The Institutional Workshop.

### WITHIN THE HOSPITALS.

### WESTMINSTER HOSPITAL.

(By Our Special Commissioner.)

Nestling near the Abbey, and occupying the best site in Broad Sanctuary, Westminster, there are few, if any, institutions, not excepting St. George's, which should command a more liberal supply of funds than the Westminster Hospital. For some cause—possibly the earnest endeavour of its committee and governors to prevent extravagance in any department—the funds so far given to this institution have been surprisingly small. It needs, and should immediately receive, an increase in its income of two thousand pounds per annum. Some years ago many thought, and still think, that it would have been better had the governors determined to remove the hospital so as to be nearer to Battersea, instead of reconstructing the building on its present site. A recent visit to the hospital, and a careful consideration of all the circumstances, lead us to feel that it is for the advantage of Londoners, and of metropolitan hospitals generally, that the Westminster Hospital has not been transplanted. This view is justified by the circumstance that the site is so restricted as to necessitate the utmost economy in space and staff, and the maximum regard to excellence of administration and hygienic completeness. Economically considered, too, the retention of the present site was wise, because it enables the committee of Westminster Hospital and its officers to show to demonstration the smallest cost at which a hospital bed can be efficiently maintained in a London hospital. We believe the authorities would be well advised to establish a school or class for training hospital secretaries and housekeepers, because there is no institution with which we are acquainted where sounder principles of economy prevail, or where the patients declare themselves to be more comfortable than at the Westminster Hospital. In these circumstances the festival dinner, which is to take place on July 6th, H.R.H. the Duke of Connaught in the chair, should yield at least £5,000, and secure an increase to the permanent income of not less than £2,000 per annum.

#### The General Aspect of the Hospital.

A visitor who is well acquainted with modern hospitals will be struck at once with the feeling that everything is here to be seen in miniature, although the hospital contains 205 beds. It must have been a difficult task for the architect to provide for modern hygienic requirements when the present buildings were reconstructed. We saw the wards and offices under the most unfavourable conditions, as the hospital is to be closed on July 1st for renovation and repair. We are able, therefore, to say, having regard to the difficulties of the site, that the architect has made the most of his small opportunities, and that apart from the drawbacks incidental to small lavatories and bath-rooms, and the lack of almost necessary room for the ward offices, the arrangements at the Westminster Hospital are sufficient. The general aspect of the wards, of the sisters and nurses, and of the patients, points to a homely feeling throughout the institution, where everybody is cheerful, happy, and reasonably contented. The sisters and nurses are businesslike and kindly. One gratefully misses the air of flightiness which is sometimes painfully visible in hospital wards where the staff is large and the buildings are scattered and straggling. At Westminster each sister and nurse was in her place, doing her business—on duty, in fact, and at "attention." Yet the atmosphere was bright and comfortable, and one felt, as old patients have often told us, that to be ill in the Westminster Hospital is to be well cared for and comfortable. Comfortable, that is to say, in the sense of contentment of mind, the assured certainty that everybody is doing their best to help the patient to recover, and that the whole place exhibits the in-dwelling, abiding presence of a Christ-like spirit, which blesses patients and nurses, and doctors, too. It seems to us that if, before next Hospital Sunday, some members of the congregation frequenting the Abbey could be induced to visit the hospital so small a collection as £200 would never be recorded again.

#### Efficiency and Economy.

The Westminster Hospital has the good fortune to be managed by an executive committee of gentlemen who take a pride in the institution, and devote much time to its administration. It follows that everything is done with care and deliberation, and that the business of no hospital in the country is conducted with greater efficiency and economy. They are fortunate in having in Mr. Sidney M. Quennell a secretary of ability and experience, whose wise judgment and invariable courtesy impress all with whom he is brought in contact. It is much that the cost per bed at the Westminster Hospital should be less than that of any other metropolitan hospital possessing a medical school. It is more, that despite this fact a close inquiry has convinced us that everything is done to give the patients all that they can possibly want of the best quality, and that nothing in the hospital is done in a mean or niggardly spirit.

We have been much impressed with the interest taken in individual patients, which finds practical expression in the endeavour to provide that no one shall leave the hospital without having a suitable place to go to, and the best chance of complete restoration to health. There can be no doubt that many people might do an infinity of good by associating themselves with individual hospitals, under the direction of the authorities, with the object of looking after those patients who have to leave the hospitals before they are completely restored to health. Here is a field which might be fruitful in results to the poor if a few earnest people with time and means would devote themselves to its cultivation.

#### A Few Points of Interest.

We noticed, in the course of our inspection, that there is a play-room for children, that the children on the surgical side are treated in the general wards, whilst a special ward is set apart for children suffering from medical diseases. The condition of the children was excellent, and a happier set of hospital patients than those on the surgical side we have never seen anywhere. In the medical ward the majority of the children were surprisingly young, a circumstance which says much for the kindness of heart of those responsible for their admission, whilst it points to a condition of affairs among the poorer homes of Westminster which should excite interest, with a view to finding a speedy remedy. Flowers there were in abundance, and we were glad to learn that the wealthy residents in the neighbourhood display a conspicuous liberality to the Hospital in this respect; we were told, indeed, that even at those seasons of the year when flowers are most scarce they are never wanting in more or less profusion in the wards of Westminster Hospital. There is an excellent chapel situated in the centre of the hospital, and it was very pleasant to hear the voices of the choir boys, which could be heard by the patients in the wards, and which must have had a most soothing and welcome effect upon them. The out-patient department, having regard to the site, has been well planned. The patients are made to circulate so as to prevent any crowding or delay. Many of the arrangements exhibited forethought and interest worthy of commendation. We observed a notice suspended on the walls of the out-patient department, calling patients' attention to the fact that, if they could afford it, they should belong to a provident dispensary, or employ a private medical attendant. We further observed an ingenious call bell within reach of the physician in attendance, which enables him to summon the patient, nurse, or porter without moving from his seat, and which might usefully be adopted in other hospitals. The authorities of the Westminster Hospital are wisely endeavouring to secure subscriptions from the less well-to-do inhabitants in small sums of 5s. or 10s. 6d. per annum. We believe that this new departure will yield at least half of the £2,000 of additional income that the Westminster Hospital now needs. Although the wards are small; although the sisters and nurses are sometimes hard pressed for necessary room; although the whole site is restricted, still, we repeat that the retention of the Westminster Hospital on its present site was a wise step. It must have an excellent educational effect upon the administration of every metropolitan hospital, where the tendency to extensive buildings entails much greater outlay for maintenance, and makes it more difficult to maintain discipline and regularity. It is a genuine pleasure to state that the administration of the Westminster Hospital is one of the most business-like and kindly in the United Kingdom.

